# Negative symptoms and social and occupational functioning differentiate systematic paraphrenia from schizophrenia: results from a cross-sectional study

**DOI:** 10.1192/j.eurpsy.2022.2052

**Published:** 2022-09-01

**Authors:** L.A. Fernandes, B. Trancas, T. Maia, N. Borja Santos

**Affiliations:** Hospital Prof. Doutor Fernando Fonseca EPE, Psychiatry, Amadora, Portugal

**Keywords:** paraphrenia, schizophrénia, negative symtpoms, Psychosis

## Abstract

**Introduction:**

Kraepelin’s systematic paraphrenia (SP) has been historically used to identify a group of patients in the psychosis-spectrum with good global functioning and reduced impairment in volition and emotions.

**Objectives:**

Cross-sectional study comparing a group of patients with SP with another with schizophrenia (SZ).

**Methods:**

We consecutively recruited SP cases from a single centre. SZ cases were selected to match those in the SP group in terms of age and sex. We diagnosed SP using the Munro Criteria and SZ using ICD-10. We collected standard sociodemographic and clinical data. All patients were under follow-up in a community mental health team at the time of the study. We used PANSS total score (PANSS-TS) to assess disease severity and its subscales to evaluate positive (PANSS-P) and negative (PANSS-N) symptoms, and general psychopathology (PANSS-GP). We applied SOFAS to assess social and occupational functioning.

**Results:**

We recruited 32 patients, 16 with a diagnosis of SP and 16 with a diagnosis of SZ. The two groups did not differ in terms of sociodemographic data. SP cases showed lower values for PANSS-TS (SP: mean=51.63±12.49; SZ=77.76±14.12; p<0.001), PANSS-NS (SP: mean=15.50±5.97; SZ: mean=26.06±5.39; p<0.001), and PANSS-GP (SP: mean=24.31±5.51; SZ: mean=37.13±5.62; p<0.001). Groups did not differ in terms of positive symptoms. SOFAS scores were significantly higher in SP (SP: median=68, interquartile range (IQR)=19; SZ: median=41, IQR=24; p<0.01). PNSS-NS negatively correlated with SOFAS only in the SP group (r=-0.716; p=0.002).

**Conclusions:**

SP differs from SZ in negative symptoms and social and occupational functioning. These findings suggest clinical features can differentiate SP from SZ.

**Disclosure:**

No significant relationships.

